# QTL detection for *Aeromonas salmonicida *resistance related traits in turbot (*Scophthalmus maximus*)

**DOI:** 10.1186/1471-2164-12-541

**Published:** 2011-11-02

**Authors:** Silvia T Rodríguez-Ramilo, Miguel A Toro, Carmen Bouza, Miguel Hermida, Belén G Pardo, Santiago Cabaleiro, Paulino Martínez, Jesús Fernández

**Affiliations:** 1 Departamento de Bioquímica, Genética e Inmunología, Facultad de Biología, Universidad de Vigo, 36310 Vigo, Spain; 2 Departamento de Producción Animal, ETS Ingenieros Agrónomos, Universidad Politécnica de Madrid, Ciudad Universitaria, 28040 Madrid, Spain; 3 Departamento de Genética, Facultad de Veterinaria, Universidad de Santiago de Compostela, 27002, Lugo, Spain; 4 Cluster de la Acuicultura de Galicia (CETGA), A Coruña, 15965, Spain; 5 Departamento de Mejora Genética Animal, Instituto Nacional de Investigación y Tecnología Agraria y Alimentaria, Ctra. A Coruña Km. 7.5, 28040 Madrid, Spain

## Abstract

**Background:**

Interactions between fish and pathogens, that may be harmless under natural conditions, often result in serious diseases in aquaculture systems. This is especially important due to the fact that the strains used in aquaculture are derived from wild strains that may not have had enough time to adapt to new disease pressures. The turbot is one of the most promising European aquaculture species. Furunculosis, caused by the bacterium *Aeromonas salmonicida*, produces important losses to turbot industry. An appealing solution is to achieve more robust broodstock, which can prevent or diminish the devastating effects of epizooties. Genomics strategies have been developed in turbot to look for candidate genes for resistance to furunculosis and a genetic map with appropriate density to screen for genomic associations has been also constructed. In the present study, a genome scan for QTL affecting resistance and survival to *A. salmonicida *in four turbot families was carried out. The objectives were to identify consistent QTL using different statistical approaches (linear regression and maximum likelihood) and to locate the tightest associated markers for their application in genetic breeding strategies.

**Results:**

Significant QTL for resistance were identified by the linear regression method in three linkage groups (LGs 4, 6 and 9) and for survival in two LGs (6 and 9). The maximum likelihood methodology identified QTL in three LGs (5, 6 and 9) for both traits. Significant association between disease traits and genotypes was detected for several markers, some of them explaining up to 17% of the phenotypic variance. We also identified candidate genes located in the detected QTL using data from previously mapped markers.

**Conclusions:**

Several regions controlling resistance to *A. salmonicida *in turbot have been detected. The observed concordance between different statistical methods at particular linkage groups gives consistency to our results. The detected associated markers could be useful for genetic breeding strategies. A finer mapping will be necessary at the detected QTL intervals to narrow associations and around the closely associated markers to look for candidate genes through comparative genomics or positional cloning strategies. The identification of associated variants at specific genes will be essential, together with the QTL associations detected in this study, for future marker assisted selection programs.

## Background

In aquaculture, disease resistance related traits are of particular importance. The reason is that interactions between fish and pathogens, that may be harmless under natural conditions, often result in disease problems in aquaculture systems because of the added stress from biological, physical and chemical factors [1]. This is especially important due to the fact that, in contrast to farm animals, the strains used in aquaculture usually have been very recently derived from wild strains [2] and, therefore, have had little time to adapt to the new disease pressures within the aquaculture environment.

Improvements in the performance of any productive or physiological characteristic of the cultured species can be achieved (if the trait is genetically determined) through artificial selection. Several breeding programmes have been developed for different traits in aquaculture species usually involving growth rate, cold tolerance and disease resistance. However, implementing classical breeding programs focused on disease resistance traits could be highly problematic, since the phenotypic measurement of these traits is often complex and expensive. It is not possible to evaluate specimens to be selected and thus, evaluation has to be performed on relatives. Moreover, the required challenges may cause animal suffering and increase risks of infection at farm facilities [3]. Despite those disadvantages, some populations of Atlantic salmon have been already selected for resistance to bacterial and viral diseases. Selection for resistance to Infectious Pancreatic Necrosis (IPN) virus, based on bath challenge tests of several hundred families of first feeding fry, showed 66.6% and 29.3% mortality for low and high resistant strains, respectively [4]. Also, high resistance to IPN in rainbow trout was achieved by selective breeding [5]. In that study, the commercial strain RT-201 artificially challenged with IPN virus showed a mortality of 4.3%, whereas the highly sensitive controls reached 96.1%. In carps, Schäperclaus [6] found resistance to the dropsy disease, selected lines suffering low mortality (11.5%) well below the unselected ones (57%).

Positive response to selection pressures is possible because resistance against particular diseases affecting aquaculture species often shows moderate to high heritabilities and, thus, there is a large potential for genetic improvement. For example recent heritability estimates for resistance to *Aeromonas salmonicida *ranged from 0.43 to 0.62 in Atlantic salmon [reviewed by [7]], and it was 0.51 ± 0.03 in *Salvelinus fontinalis *[8].

Turbot (*Scophthalmus maximus*) is a flatfish that has been intensively cultured during the last decade due to its great commercial value. Its production in Europe has increased from 3000 Tm in 1996 to 9246 Tm in 2009 [9]. Increasing growth rate, controlling sex ratio (females largely outgrow males) and enhancing disease resistance currently constitute the main goals of genetic breeding programmes in this species. Pathologies constitute one of the main problems of turbot culture. Among these, furunculosis, caused by *Aeromonas salmonicida*, has produced important losses to turbot industry [10,11]. Genomic resources of turbot have increased in the last years [12,13] and an immune-enriched oligo-microarray was designed and applied to identify candidate genes of resistance to *A. salmonicida *[14]. Also, a microsatellite consensus genetic map including centromere positions was reported in this species [15,16], and recently, 31 EST-linked microsatellites, particularly useful for comparative genomics, were added to the consensus map [17]. Combining functional genomics strategies with the detection of genomic regions associated to productive characters (using genetic maps) increases the power to identify genes involved in the phenotypic differences occurring within and between families.

Besides an infinitesimal component (due to small effects of a huge number of loci) the variation in quantitative traits may be also controlled by a few genes with larger effects. Genomic regions closely linked to those genes show association with the trait phenotype and are known as quantitative trait loci (QTL) [18]. The effects of allele segregation at molecular markers throughout the genome can be used to determine the number and position of trait-related QTL, as well as the magnitude of their effects [19]. If, eventually, the responsible gene of a large effect on a trait is detected and the causal mutation determined, selection could be exerted directly on the genotype for that locus (Gene Assisted Selection, GAS). Alternatively, genetic maps provide DNA markers tightly linked to genes affecting different traits. Such markers can be used in Marker-Assisted Selection (MAS), selection based partly or fully on DNA marker genotypes. Consequently, disease resistance traits are candidates for the implementation of MAS and, especially, GAS programs, which would allow the evaluation of individuals without exposing them to the pathogen or relying on relatives' information alone.

There have been a certain number of studies on disease resistance QTL in the main aquaculture species, especially focused in rainbow trout and Atlantic salmon within fish. In rainbow trout, several QTL were detected for resistance to IPN and IHN (Infectious Hematopoietic Necrosis) viruses [20-23] and to different parasites, including *Ceratomyxa shasta *and *Myxobolus cerebralis *[24,25]. The identified QTL in response to IHN virus were detected in more than one family supporting their consistency [22], and in the case of resistance to *Myxobolus cerebralis*, a very strong association was detected which explained between 50 and 86% of the phenotypic variance across families [25]. In Atlantic salmon, QTL for disease resistance have been reported for the ISA (Infectious Salmon Anaemia) virus [26], for the bacterium *Aeromonas salmonicida *responsible of furunculosis [27] and for the ectoparasite *Gyrodactylus salaries *[28]. Of particular relevance in this species was a recent study on ten full-sib families for mapping QTL for resistance against the IPN virus in post-smolts of Scottish origin, based on data from a field trial [29]. In this study, a major QTL explained up to 21% of the phenotypic variation in the data set and was found to segregate in 7 out of 20 parents investigated. Additionally, this QTL mapped to the same location of a recently detected QTL for IPN-resistance that explained 29% of the phenotypic variance using ten large full-sib families of challenge-tested Norwegian Atlantic salmon. This particular QTL was found to be segregating in 10 out of 20 parents, and a subsequent fine-mapping with additional markers narrowed the QTL peak to a 4 cM region on linkage group 21 [3]. This QTL, detected in two different populations, is now being implemented in within-family selection in both Scotland [30] and Norway [3]. QTL for disease resistance have also been reported in a few cases in non-salmonid teleosts, including those for stress/immune response in tilapia [31], for resistance to pasteurellosis in gilthead sea bream [32] and for resistance to lymphocystis viral disease in Japanese flounder [33]. Other QTL for disease resistance traits in aquaculture species were identified in *Crassostrea virginica*, *Ostrea edulis *and *Paralichthys olivaceus *[see 7 for a review].

To date, only two studies identified QTL in turbot. One detected a QTL for body length highly associated with the marker YSKr51, explaining 12.4% of the phenotypic variance [34], and the other one identified a significant sex-determining QTL highly associated with the SmaUSC-E30 marker, which allowed for correct sex determination in up to 98.4% of the studied individuals [35]. The construction of a genetic map with appropriate marker density is necessary to detect QTL controlling quantitative traits of economic interest in aquaculture [20]. However, genetic linkage maps of most aquaculture species have only recently been available. Bouza et al. [17] reported an updated consensus including a total of 273 microsatellites clustered in 26 linkage groups, comprising 1343.2 cM length, with an average distance between markers of 6.5 ± 0.5 cM.

In the present work, a genome scan for QTL affecting resistance and survival to *A. salmonicida *in four turbot families was carried out using the reported microsatellite panel. The objectives were: (1) to locate QTL on the available linkage map, (2) to compare QTL obtained through the use of two different methodologies: linear regression and maximum likelihood, and (3) to determine the association between markers and traits.

## Methods

### Families

Four full-sib turbot families were used to identify QTL for resistance and survival to *A. salmonicida *and to evaluate the association of those traits with the genotyped markers. Three of these families were obtained from the *Stolt Sea Farm S.A*. breeding program (*FamAS-1*, *FamAS-2 *and *FamAS-3*) and one family was obtained from *Insuiña S.A*. (*FamAP*). Both companies have their facilities located in NW Spain.

Since no selected strains for high and low disease resistance exist in turbot, families were selected trying to emphasize genetic divergence between parents to detect as much as possible allelic variants associated with resistance. Thus, families founded with unrelated grandparents and, when possible, from different Atlantic origins, were chosen. Also, we selected families where a three-generation pedigree was available. This enabled us to know the linkage-phase between markers for a more consistent statistical analysis. This process finally led to four independent full-sib pair analyses.

### Trait measurement

All offspring of each family (approximately 150 individuals per family) were intracelomically injected at the age of four months (mean weight around 20 g) with a highly virulent *A. salmonicida *strain. Although *A. salmonicida *infection was previously reported to last around 21 days [11], the experiment was prolonged to 39 days to increase phenotypic variance for survival. After injection, two disease resistance related traits were evaluated: resistance (*Re*) and survival (*Su*). Around one hundred individuals for each family were selected to evaluate both traits (the 50 most resistant and the 50 most sensitive individuals). The dichotomous trait *Re *was defined as the survival *vs *non survival status of individuals at the end of the experiment (i. e. day 39 after the injection). The trait *Su *was defined as the number of days one individual survived (i.e. elapsed days until the individual died or the experiment finished). Consequently, this is a censored trait since all individuals still alive at the end of the study were scored with the same value for survival.

### Genetic map

The panel of markers used for QTL identification was reported by Martínez et al. [35] and it is based on the consensus map by Bouza et al. [15], the new EST-linked microsatellites by Bouza et al. [17] and the centromere mapping [16].

Table 1 shows the number of analyzed microsatellites, the number of linkage groups, the map length, the average distance between microsatellites and the average number of microsatellites per linkage group. The average distance between markers in the map ranged between 15.53 and 16.06 cM, being below the minimum distance proposed for QTL detection (< 20 cM) [36].

**Table 1:** Screening figures in the four families analyzed for QTL identification

Family	NM	LG	MLe	D	NMG
*FamAS-1*	104	22	1147.80	15.62	4.73
*FamAS-2*	98	22	1105.10	15.53	4.46
*FamAS-3*	99	22	1132.30	15.90	4.50
*FamAP*	101	23	1149.40	16.06	4.39

NM: Number of markers; LG: linkage groups; MLe: map length in cM; D: average distance between markers in cM; NMG: mean number of microsatellites per linkage group.

### QTL analyses

Two programs were used to detect QTLs: GridQTL [37] and QTLMap [38]. Within both methodologies, two approaches were followed. First, a single QTL was assumed at each LG. Afterwards, a two-QTL model was also tested within each LG.

#### GridQTL

This software http://www.gridqtl.org.uk/ implements a linear regression (LR) methodology, considering the linkage phase between markers according to pedigree information. The default regression method [39] was applied, and the genome and chromosome-wide significance thresholds were estimated by implementing bootstrapping at *p *= 0.05 and 0.01 [40], with a permutation test of 10, 000 iterations [41].

#### QTLmap

This software https://qgp.jouy.inra.fr/index.php?option=com_content&task=view&id=17&Itemid=28 detects QTL through interval mapping using a maximum likelihood (ML) test. To determine the significance level 10, 000 simulations were performed for each trait and LG, with a heritability set to 0.10 [42].

For both methodologies, an outbred full-sib model was used and a QTL was considered suggestive when significance was between 5% and 1% at chromosome-wide level, and significant when significance was below 1% at chromosome-wide level or when significance was below 5% at genome-wide level [43]. These thresholds also allow establishing a confidence interval to allocate the detected QTL.

Sex was not included into the model because it was not available. Although there is some debate about the influence of growth-related traits on the resistance to diseases (see for example [44] for positive results, and [45] and [46] for absence of correlations between both types of traits), weight and length were included as covariates within the model to reduce the stochasticity.

### Association analyses

A one way analysis of variance (ANOVA) was performed on the phenotypic values (resistance and survival) of the progeny for each family using individual genotypes from markers within the LGs where a significant or suggestive QTL was found. The objective was to detect associations between markers and traits by estimating the between-genotype component of the observed phenotypic variance (i.e. differences attributable to the different marker genotypes). To avoid false positives due to multiple testing, a simple Bonferroni correction was performed for all tests involving those markers within the same LG. Each ANOVA also provided a corrected *R^2 ^*value useful to estimate the reduction of the overall phenotypic variance of the trait due to the model fitting, thus providing the proportion of the trait variance predictable from the given marker genotypes.

## Results and Discussion

### Trait values

Table 2 shows the number of observations and the mean for the traits recorded (resistance and survival) in the four families of the experiment. The standard deviation ranged between 13.12 - 15.31 days for survival and 0.44 - 0.50 for resistance. Notice that a greater power for detection is expected in challenges where there is approximately a 50% of survival (LD_50_), as it occurred in *AS *families. Although a lower survival (26%) was observed for family *AP*, this did not preclude the detection of QTL in that family (see Table 3). Mean weight and length ranged between 31.62 - 46.45 g and 10.70 - 12.83 cm, respectively.

**Table 2:** Statistics of the measured traits in the four families analyzed (± standard deviation)

Family	Weight (g)	Length (cm)	Trait	*N*	Mean
*FamAS-1*	46.45 ± 8.49	12.83 ± 0.78	*Re* *Su*	100 100	0.5 24.44
*FamAS-2*	26.69 ± 6.00	10.70 ± 0.77	*Re* *Su*	100 100	0.5 24.17
*FamAS-3*	31.62 ± 7.18	11.33 ± 0.86	*Re* *Su*	100 100	0.5 23.58
*FamAP*	32.13 ± 5.20	11.43 ± 0.59	*Re* *Su*	113 113	0.26 17.04

*Re *= resistance; *Su *= survival; *N *= number of individuals.

**Table 3:** Location of the QTL detected for resistance and survival to *A. salmonicida *with two different statistical methodologies

		Resistance						Survival					
		
		LR			ML			LR			ML		
**Family**	**LG**	**EP**	**Interval**	**Sig**.	**EP**	**Interval**	**Sig**.	**EP**	**Interval**	**Sig**.	**EP**	**Interval**	**Sig**.

*FamAS-2*	4	0	0 - 11	*	10	0 - 18	s	0	0 - 15	s	10	0 - 19	s
	11							43	37 - 44	s			
	16				49	46 - 49	s				49	41 - 49	s
													
*FamAS-3*	1										1	0 - 8	s
	6	0	0 - 42	*	26	0 - 66	**	0	0 - 43	*	26	0 - 66	**
	9				66	64 - 66	s						
	12	52	49 - 52	s	52	50 - 53	s	52	51 - 52	s	52	50 - 53	s
													
*FamAP*	5				21	5 - 36	*				19	10 - 29	**
	9	31	27 - 35	*	38	29 - 45	*	31	24 - 37	*	36	31 - 40	**
	13	0	0 - 4	s				0	0 - 4	s			
	16				35	21 - 49	s				36	23 - 55	s
	18										16	12 - 26	s

LR: linear regression; ML: maximum likelihood; LG: linkage group; EP: estimated position (in cM); Interval: confidence range of the detected QTL (in cM); Sig.: significance level; QTL was considered suggestive (s) when significance was between 5% and 1% at chromosome-wide level, and significant when significance was below 1% at chromosome-wide level (*) or when significance was below 5% at genome-wide level (**).

### QTL analyses

Table 3 shows the location of the detected QTL (LG, estimated position and interval) with both methodologies (LR and ML). There were no QTL at genome-wide level with the LR approach, but two suggestive (LG12 and LG13) and three significant (LG4, LG6 and LG9) QTL were detected for resistance, and four suggestive (LG4, LG11, LG12 and LG13) and two significant (LG6 and LG9) QTL were detected for survival at chromosome-wide level. Using the ML methodology, one QTL for resistance (LG6) and three for survival (LG5, LG6 and LG9) were identified at genome-wide level with significance below 1%. Additionally, three suggestive (LG4, LG12 and LG16) and two significant (LG5 and LG9) QTL for resistance, and five suggestive (LG1, LG4, LG12, LG16 and LG18) QTL for survival were detected at chromosome level.

In our study, four full-sib families from segregating populations were evaluated separately using two different methodologies. Kao [47] investigated the performances of LR and ML methods for QTL detection, suggesting the application of LR as an initial procedure to obtain preliminary results and then use the ML method as a final procedure in order to obtain the most conclusive results. Our data support in part Kao's suggestion, since the highest significance levels were yielded by ML for the same QTL detected with both methodologies. The concordance in QTL location between both methods or between different families (LG16 in *FamAS-2 *and *FamAP*; see Table 3) increases the confidence of the results obtained.

QTL were less significant when length and weight were excluded from the model (data not shown) indicating that body size may contribute to or may be correlated with another trait affecting disease resistance. The regression coefficients for the covariates themselves were not significant except for body weight in one of the families (*FamAP*). Overturf et al. [44] indicated a positive correlation between body size and disease resistance related traits, but other authors detected no significant correlation between both types of traits [45,46]. Although the inclusion of body size improved QTL detection in our study, the exact relationship with resistance to *A. salmonicida *is not known.

Classical QTL mapping methods assume that traits follow a normal distribution [48]. However, the categorical (dead or alive) and survival (length of life) data used in this study are not-normally distributed. For such traits, classical QTL detection methods could have a lower power and a bias when estimating the effects and position of QTL. Nevertheless, it has been proposed that classical interval mapping methods using a Gaussian model on censored data, analyzed as if they were uncensored, have not a reduced accuracy on QTL location and estimation of QTL effects [49].

As can be seen in Table 3, several QTL were detected for resistance and survival at close positions within the same LG. One reason for this agreement could be the high relationship expected between both traits, since the resistance concept also includes survival (resistant individuals survive for a long period, in fact the whole experiment). Other reason could be that the same genes were involved in the mechanisms underlying both disease resistance traits.

The power to detect QTL depends on the heritability of the trait, the recombination distance between QTL and markers, the proportion of phenotypic variance explained by the QTL, the QTL allele frequency and the sample size [50]. In addition, the power of QTL analysis is limited, since only QTL segregating in one or both parents can be detected. In fact, there were no QTL detected within *FamAS-1*. Considering that families come from unrelated and genetically divergent grandparents from natural populations of the Atlantic area, identified QTL in our study could be representative of the genetic architecture of disease resistance related traits in turbot. Anyway, the detected QTL should be verified in other turbot families before their use in breeding programs.

The probability of success and the accuracy of results of QTL studies depend on the precision and density of the available genetic map. Following this idea, the map used in this study is being further refined and, thus, the QTL detection could also be improved. Currently, Martínez et al. (unpublished data) are developing a consensus map comprising 463 markers (microsatellites and EST) with a moderately dense coverage.

### Association analyses

All markers within the same linkage group where a suggestive or significant QTL was detected were analyzed. Table 4 shows the results only for markers significantly associated with any of the traits. At least one significantly associated marker was found in all linkage groups where a QTL was detected, except for LGs 1, 12, 13 and 18. However, within these LGs only suggestive QTL were detected (see Table 3). Remarkably, not always the most significantly associated markers were the closest to the estimated position of the QTL (data not shown). The reasons could be: (1) low information content of the closest marker due to the parents genotype; (2) a large extension of the area in linkage disequilibrium with the detected QTL, which could result in positive associations between the trait and the genotypes at several markers; (3) the existence of a secondary segregating QTL, although this situation was not detected when LGs were analyzed for the possibility of carrying two QTL; and finally (4) marker positions in the map are not definitive, which could modify the order and the distance between markers. For example, it should be noticed that the present genetic map of turbot has more linkage groups (26) than chromosomes observed in this species (2*n *= 44 chromosomes) [51].

**Table 4:** Proportion of the phenotypic variance explained by the markers significantly associated with the evaluated traits

**Trait**^**1**^	Family	LG	Method^2 ^and QTL position (cM)	**Marker**^**3**^	Marker **position (cM)**^**4**^	*R^2 ^*(%)
*Re*	*FamAS-2*	4	LR: 0/ML: 10	Sma-USC47	0.00	7.4
						
	*FamAS-3*	6	LR: 0/ML: 26	Sma-USC147	4.52	16.2
						
	*FamAP*	5	ML: 21	SmaUSC-E30	0.00	8.9
		9	LR: 31/ML: 38	SmaUSC-E23	30.75	10.5
				SmaUSC-E41	45.68	12.4
				Sma-USC21	53.91	11.6
		16	ML: 35	Sma-USC256	32.75	13.6
						
*Su*	*FamAS-2*	4	LR: 0/ML: 10	Sma-USC47	0.00	8.1
		11	LR: 43	Sma-USC158	23.39	10.1
						
	*FamAS-3*	6	LR: 0/ML: 26	Sma-USC147	4.52	16.8
						
	*FamAP*	5	ML: 19	SmaUSC-E30	0.00	8.9
		9	LR: 31/ML: 36	SmaUSC-E23	30.75	12.3
				SmaUSC-E41	45.68	12.8
				Sma-USC21	53.91	12.0
		16	ML: 36	Sma-USC256	32.75	12.4

*^1^Re*: resistance; *Su*: survival.

LG: linkage group.

^2^LR: linear regression method; ML: maximum likelihood method;

^3^BLAST annotation [17] was only recorded for Sma-USC23 (3-Hydroxibutirate dehydrogenase type 2) and Sma-USC41 (Helicase with zinc finger domain).

^4^Marker position in centimorgan (cM) within the genetic map by Bouza et al. [17].

*R^2 ^*(%): proportion of the explained phenotypic variance.

Markers showing significant association with studied traits explained from 7 to 17% of the phenotypic variance. The marker SmaUSC-E30, significantly associated with resistance and survival traits at LG5, was previously associated to the major QTL for sex determination in turbot [35]. This may suggest an influence of sex in the measured traits that could be related either to the reported growth rate differences between sexes in turbot [52] or to sex-susceptibility/immune differences [53]. A relationship between growth and immune function was previously reported in fish [54,55]. In the case of LG9, up to three markers showed significant association with both traits (Table 4: SmaUSC-E23, SmaUSC-E41 and Sma-USC21), the two first ones being functionally annotated as immune-related genes [14].

A high explained phenotypic variance was also observed for an IPN-resistance QTL in salmonids 21% [29] and 29% [3]. A QTL for ISA-resistance, also in Atlantic salmon, explained 6 - 9% of the phenotypic variance [26]. In rainbow trout two QTL for IPN-resistance were identified explaining 27 and 34% of the phenotypic variance, respectively [20].

Several associations have been detected in teleosts between disease resistance and candidate genes, mostly involving polymorphism of the Major Histocompatibility Complex (MHC), an essential gene family for adaptive immunity [7]. In rainbow and cutthroat trout, an association was detected with the resistance to IHN virus [56]; in rainbow trout, a suggestive association with the resistance to Bacterial Cold Water Disease (BCWD) [57]; and in Atlantic salmon, associations were detected for resistance to *A. salmonicida *[27,58,59], IHN [60], and ISA [27]. Beyond salmonids, associations between MHC markers and disease resistance have also been reported in other important farmed teleosts like Japanese flounder (resistance to *Vibrio anguillarum*) [61]; common carp (resistance to *Cyprinid Herpesvirus*-3) [62], and in the turbot (resistance *to Edwardsiella tarda*) [63]. Several of these associations involved the MHC class IIB gene, an observation which strongly recommends the mapping of this gene in turbot to cross this information against the QTL locations from this study and to evaluate its possible role in general resistance to bacterial pathogens. Nevertheless, direct selection on MHC variants could be not advisable since balancing selection [57] or overdominance [64] have been suggested to be acting on this polymorphism. The risk of pathogen-specific allelic selection could determine undesired loss of MHC variation, which may increase susceptibility to other pathogens. Furthermore, it has been highlighted that other genes may also play an important role to explain genetic variance for disease resistance [7]. Recently, significant associations with other relevant immune-genes have been reported in common carp (Interleukin 10 (IL-10) with resistance to *cyprinid herpesvirus-3 *[65]) and in grass carp (Toll-like Receptor-3 (TLR3) with resistance to reovirus [66]).

A revision and a refinement of the mapping regions where QTL were located should be performed to further narrow the intervals and facilitate the advance on candidate gene strategies. Also, mapping of differentially expressed (DE) genes identified in response to *A. salmonicida *infections [14] should be evaluated or acquired to cross this information with QTL position. None of the immune-related genes significantly associated with resistance and survival traits in this study (SmaUSC-E23, SmaUSC-E41) were found to be regulated in response to *A. salmonicida *infection [14]. Nevertheless, two DE genes detected in response to this pathogen [14] were located in the vicinity of two QTL at LG9 and LG11, respectively. SmaUSC-E41, linked to the immune-related DE ecdysone receptor A (BLAST annotation; E-value: 3E^-8^), was located at LG9 close to the marker SmaUSC-E5 [4 cM; see [17]]. This gene pertains to a nuclear receptor superfamily present in all Metazoa, containing ligand dependent transcription factors related to immune system and regulation of inflammatory processes [67]. On the other hand, Sma-USC147 was 7 cM apart from the significantly associated SmaUSC-E24 marker at LG11. This marker is linked to a DE gene (annotated as PRA1 family protein 3 using BLAST; E-value: 3E^-9^), which modulates antiapoptotic activity and immune response [68]. Since these DE markers were not included in the panel of markers for QTL screening in this work, further association analysis across the same QTL families should be carried out. Finally, markers linked to relevant DE genes in the turbot and previously associated with disease resistance in other fish (e.g., TLR3, MHC class IA and MHC IIA) [14,66,7], should be mapped. This would enable to test their association within families to provide new candidates for a functional explanation of resistance to *A. salmonicida *in the turbot.

If the responsible gene and the causal mutation were detected, then, GAS could be performed. Increasing map density should facilitate the detection of more closely linked markers useful to implement MAS. For both strategies, another promising approach is the use of comparative genomics [69]. A study of comparative mapping by Bouza et al. [15] identified syntenic relationships between sequences of turbot and the model fish *Tetraodon nigroviridis *(Tni): between turbot LG16 and Tni chromosome 19; between turbot LG5 and Tni1; and between turbot LG6 and Tni13. Since in the present study QTL at LG5, LG6 and LG16 were detected, the analysis of genes located at these syntenic blocks in *T. nigroviridis *through comparative genomics tools with the turbot DE genes in response to *A. salmonicida *[14] could provide relevant information about candidates underlying the detected QTL.

Finfish aquaculture is still relatively in its first stages in the large-scale animal production sector. However, it can take advantage on the vast amounts of genomic data currently being generated for livestock, as well as for model fish organisms. Many livestock genomics programs have focused on identifying genes influencing similar economic traits as those important in aquaculture, such as growth rate, disease resistance and meat quality traits. Through comparative genomic approaches, sequence information from other vertebrates can be used to quickly isolate homologous genes in finfish. It could be also interesting to extend this study to other pathogens for detection of disease resistance related QTL affecting turbot aquaculture systems in order to investigate more general disease resistance related traits.

## Conclusions

Regions controlling resistance-related traits to *Aeromonas salmonicida *in turbot were detected. Concordance on the detection of QTL between statistical methods gives more consistency to our results. A finer mapping will be necessary on those linkage groups with effect on disease resistance by increasing the density of markers on the position where the QTL were detected. In addition, functional and comparative genomics strategies could be used to confirm the detected QTL and to look for candidate genes. Alternatively, the associated markers could be useful in programs for marker-assisted selection in turbot.

## Authors' contributions

STRR was responsible of the final version of QTL and marker analysis and the writing of the article. MAT and JF were responsible of the supervision of the QTL and marker analysis and also of the supervision of the article. CB, MH and BGP performed genotyping at all families and contributed to discussion on association analysis. SC was responsible of challenges of fish and measuring of phenotypic traits. PM was responsible of the work and supervised all tasks. JF was responsible for a first version of the QTL analysis. All authors read and approved the final manuscript.
